# The Mechanisms and Boundary Conditions of the Einstellung Effect in Chess: Evidence from Eye Movements

**DOI:** 10.1371/journal.pone.0075796

**Published:** 2013-10-04

**Authors:** Heather Sheridan, Eyal M. Reingold

**Affiliations:** 1 School of Psychology, University of Southampton, Southampton, United Kingdom; 2 Department of Psychology, University of Toronto at Mississauga, Mississauga, Ontario, Canada; VU University Amsterdam, Netherlands

## Abstract

In a wide range of problem-solving settings, the presence of a familiar solution can block the discovery of better solutions (i.e., the Einstellung effect). To investigate this effect, we monitored the eye movements of expert and novice chess players while they solved chess problems that contained a familiar move (i.e., the Einstellung move), as well as an optimal move that was located in a different region of the board. When the Einstellung move was an advantageous (but suboptimal) move, both the expert and novice chess players who chose the Einstellung move continued to look at this move throughout the trial, whereas the subset of expert players who chose the optimal move were able to gradually disengage their attention from the Einstellung move. However, when the Einstellung move was a blunder, all of the experts and the majority of the novices were able to avoid selecting the Einstellung move, and both the experts and novices gradually disengaged their attention from the Einstellung move. These findings shed light on the boundary conditions of the Einstellung effect, and provide convergent evidence for Bilalić, McLeod, & Gobet (2008)’s conclusion that the Einstellung effect operates by biasing attention towards problem features that are associated with the familiar solution rather than the optimal solution.

## Introduction

During creative problem-solving, prior knowledge and experience can enhance performance by efficiently guiding us towards solutions that worked in the past. However, prior knowledge can also harm performance if the problem requires a novel solution. One of the most famous examples of the negative impact of prior experience on problem-solving is the Einstellung (mental set) effect (e.g., [1]–[5]). This effect was first demonstrated using a problem-solving task that required participants to use water jugs of known volumes to measure a specific quantity of water [1]. The participants were first shown five introductory problems that could be easily solved using a simple algorithm. Next they were shown a superficially similar problem that required a new algorithm (i.e., the “extinction problem”). Interestingly, many participants claimed that the extinction problem was insoluble, even though it was easily solved by a control group of participants who had not experienced the introductory problems. In this example, the participants’ prior experience interfered with problem-solving, because a familiar (but inappropriate) solution blocked the discovery of a new solution.

Of relevance to the present study, expertise in a domain has also been shown to induce “Einstellung-like” effects [6]–[13]. In particular, chess has proven to be a fruitful domain for investigating the mechanisms underlying the Einstellung effect (for a review, see [6]). Chess is widely considered to be an ideal experimental task for studying human cognition [14], and chess provides numerous methodological advantages, such as an interval rating scale for the measurement of chess skill [15], [16]. Capitalizing on these advantages, [8] examined the Einstellung effect in chess experts with a wide range of skill levels (Candidate Masters, Masters, and International Masters). To induce the Einstellung effect, [8] asked chess players to solve chess problems that contained both a familiar (but not optimal) solution, and a less familiar optimal solution (for a similar paradigm see [17]). Like the participants in the water-jugs experiment [1], many of the chess players failed to find the optimal solution. Importantly, [8] showed that the presence of the familiar solution reduces the performance of chess players to the level demonstrated by much weaker players (three standard deviations lower in skill level) who were given a control problem that only contained the optimal solution. Thus, the Einstellung effect can have a dramatic effect on the performance of experts in a domain-specific problem-solving situation.

Building on these findings, [7] used eye tracking to investigate the mechanisms underlying the Einstellung effect. Specifically, [7] instructed chess experts to find the fastest way to win (i.e., to find checkmate in the fewest possible moves). Replicating prior findings [8], [17], the chess experts initially discovered the familiar but longer solution (i.e., checkmate in five moves), but failed to find the shortest solution (i.e., checkmate in three moves). Importantly, the chess experts continued to look at the chess squares associated with the familiar solution, even though they reported that they were searching for alternative solutions. Moreover, as evidence that the optimal solution was not inherently difficult, a control group of chess experts successfully discovered the optimal solution when they were shown a modified version of the problem that did not contain the familiar solution. Based on this pattern of results, [7] concluded the Einstellung effect operates by biasing attention towards problem features associated with the first solution that comes to mind – thus preventing the discovery of new solutions.

**Figure 1:**
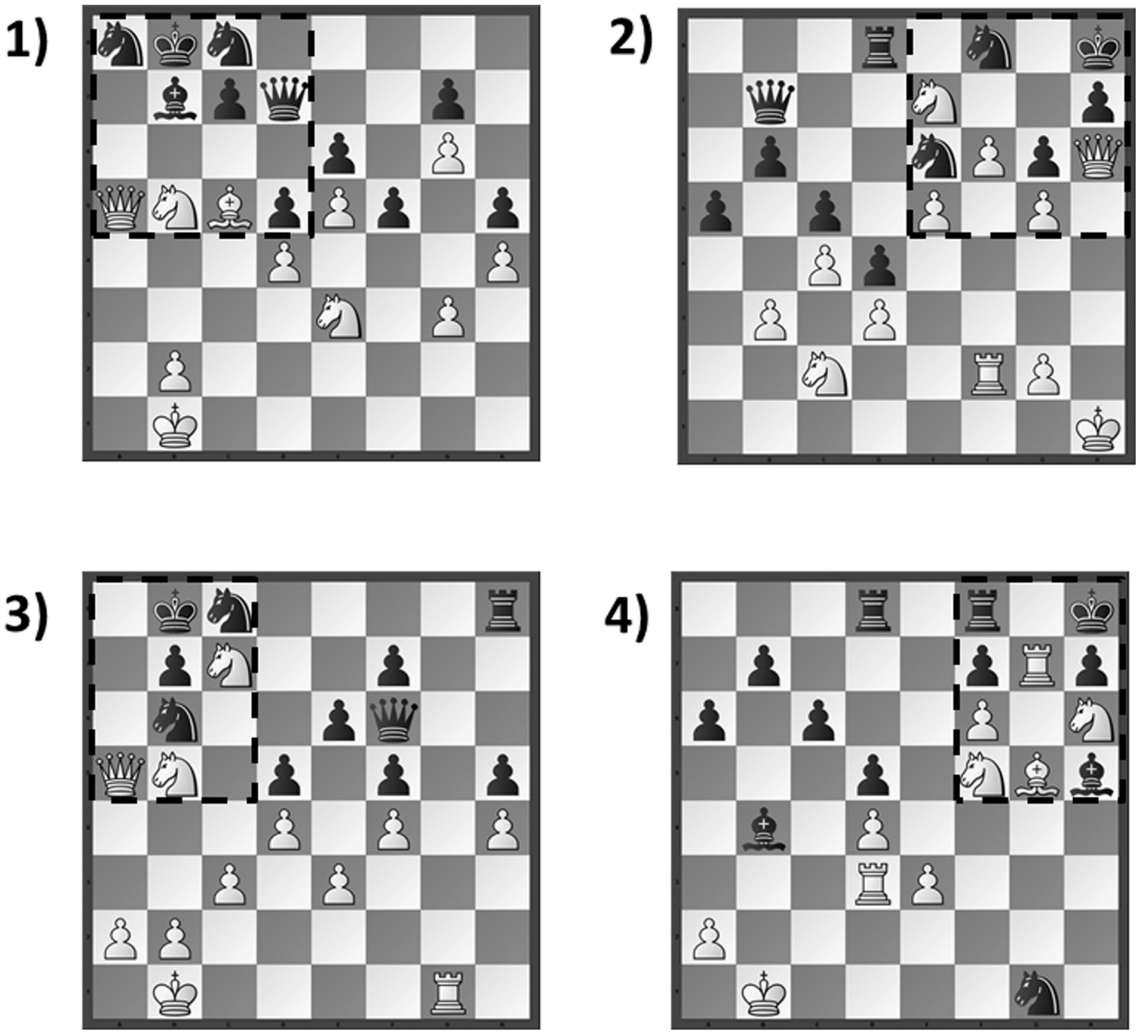
The four experimental problems (1,2,3,4). White is to move in all problems. As discussed in the text, each problem contained a familiar move (i.e., the Einstellung move) that was associated with a checkmate solution that was not possible due to the position of Black’s defenders. The Einstellung moves were always located within the target region (shown here with a dotted line). For problem 1, the Einstellung move was a reasonable move (i.e., Ba7), and for the remaining problems the Einstellung moves were blunders (i.e., Problem 2: Qg7; Problem 3: Qa7 or Qa8; Problem 4: Rg8 or Nf7). For all four problems, the optimal move on the board was located outside of the target region (i.e., Problem 1: Ng2; Problem 2: Na3; Problem 3: Rg5; Problem 4: Rb3). See Appendix S1 for further details.

Extending the investigation of [7], the goal of the present study was to further explore the bias in the spatial distribution of fixations towards locations on the chessboard that are related to the familiar but non-optimal move (henceforth, the Einstellung move). Specifically, we monitored the eye movements of both novice and expert chess players while they selected white’s best move (i.e., choose-a-move task) for a variety of chess problems that were designed to induce the Einstellung effect. As shown in Figure 1, all of these problems contained an Einstellung move that resembled a familiar checkmate solution but which was modified such that checkmate was no longer possible. The Einstellung move was located inside a target region in one corner of the board (in Figure 1 the target region is indicated with a dotted line), and there was always an optimal move located outside of the target region.

As described in Figure 1 and Appendix S1, we examined two different types of problems. The first type of problem (see Problem 1 in Figure 1) closely resembled the problems used by [8] (Experiment 2), because the Einstellung move constituted a good move that was advantageous for white, although it was not as good as the optimal move. For this type of problem, we expected to replicate [7] and [8] by showing that many of the chess players would choose the Einstellung move, rather than the optimal move. Moreover, based on the findings of [7], we expected that the chess players would have trouble disengaging their attention from the familiar solution, as shown by a high percentage of time spent fixating the target region containing the Einstellung move. In contrast, for the second type of problem, we examined a novel situation in which the Einstellung move was a blunder rather than a good move (see Problems 2, 3 and 4 in Figure 1). We expected that this change might reduce the magnitude of the Einstellung effect, such that chess players would be better able to select the optimal move, and to disengage their attention from the familiar solution. Thus, our rationale for including two different types of problems was to try to uncover boundary conditions that might modulate the strength of the Einstellung effect. In addition, we explored expert/novice differences in the magnitude of the Einstellung effect as reflected in the quality of the chosen moves and the degree to which looking behavior was biased towards the target region.

## Method

### Ethics Statement

Written informed consent was obtained from each participant (or from a parent/guardian if the participant was a minor). The research programme was approved by the Ethics Review Unit at the University of Toronto.

### Participants

Thirty-four chess players (17 experts and 17 novices) were recruited from online chess forums and from local chess clubs in Toronto and Mississauga (Canada). The mean age was 30 (range = 15 to 56 years) in the expert group, and 26 (range = 17 to 47 years) in the novice group. There was one female player in the expert group, and there were three female players in the novice group. For the expert players, the average CFC (Canadian Chess Federation) rating was 2223 (range = 1876 to 2580). All of the novice players were unrated but active club players. All of the participants had normal or corrected-to-normal vision.

### Materials and Design

The four experimental problems are shown in Figure 1. These problems were designed to give the impression that there was a familiar checkmate solution inside a target region (shown in Figure 1 with a dotted line) that was always located in one corner of the board. However, in all four problems, the checkmate solution was not possible due to the location of black’s defender pieces. For example, Problem 4 resembles the familiar “smothered mate” checkmate sequence in which a player sacrifices a valuable piece (i.e., by moving the white rook to g8) in order to draw an opponent’s piece onto a square that will block the escape route for the king (i.e., the black rook on f8 captures the white rook on g8). This checkmate solution is not possible in Problem 4 because the black bishop on h5 is protecting the f7 square, which prevents the white knight from moving to f7 to checkmate the king.

As shown in Figure 1 and Appendix S1, each problem contained one (or more) familiar moves (i.e., Einstellung moves) that were associated with the familiar checkmate solution. All of these moves involved putting the black king in check, and all of these moves were located within the target region. For Problem 1, the Einstellung move was as an advantageous but suboptimal move (i.e., Ba7), whereas for the remaining problems (i.e., Problems 2, 3 and 4), the Einstellung moves were always blunders (Problem 2: Qg7; Problem 3: Qa7 or Qa8; Problem 4: Rg8 or Nf7) that led to material loss and/or severely weakened white’s position. In all four problems, there was a better move (i.e., the optimal move) located outside of the target region (Problem 1: Ng2; Problem 2: Na3; Problem 3: Rg5; Problem 4: Rb3).

In addition to the four experimental problems, the players were shown eight filler trials that were designed to mask the purpose of the experiment. The filler trials incorporated a variety of solutions that ranged from checkmate to material gains to defensive tactics. Thus, every player completed a total of 12 problems (i.e., 4 experimental problems and 8 filler problems) that were always shown in the following trial order: two fillers, Problem 1, three fillers, Problem 2, one filler, Problem 3, two fillers, Problem 4.

### Apparatus and Procedure

Eye movements were measured with an SR Research EyeLink 1000 system with high spatial resolution and a sampling rate of 1000 Hz. The experiment was programmed and analyzed using SR Research Experiment Builder and Data Viewer software. Viewing was binocular, but only the right eye was monitored. A chin rest and forehead rest were used to minimize head movements. Following calibration, gaze-position error was less than 0.5°. The chess problems were presented using images (755×755 pixels) that were created using standard chess software (Chessbase 11). These images were displayed on a 21 in. ViewSonic monitor with a refresh rate of 150 Hz and a screen resolution of 1024×768 pixels. Participants were seated 60 cm from the monitor, and the width of one square on the chessboard equaled approximately 3.4 degrees of visual angle.

Prior to the experiment, the participants were instructed to choose white’s best move as quickly and as accurately as possible, and they were told that they would be given a maximum of 3 minutes to respond to each problem. At the start of each trial, the participants were required to look at a fixation point in the center of the screen, prior to the presentation of the chessboard. The participants were asked to press a button as soon as they had made their decision, and they then reported their move verbally to the experimenter. If three minutes elapsed prior to the button press (this occurred on 10% of the experimental trials for the novices, and 0% of trials for the experts), then the chessboard was removed from the screen and the chess player was prompted to immediately provide their best answer. At the end of the experiment, we interviewed both the experts and novices to obtain retrospective subjective responses concerning their problem-solving strategies. Specifically, we provided the chess players with a picture of each of the four experimental problems (with a dotted line surrounding the target region), and we asked them to try to recall their thought processes with regards to the target region of the board.

## Results

Our main goal was to explore the impact of the level of expertise of the chess players (i.e., expert versus novice) and the type of Einstellung problem (i.e., suboptimal versus blunder) on the magnitude of the Einstellung effect. Accordingly, in the analyses below, we assessed the magnitude of the Einstellung effect by examining the quality of the chosen moves, and the degree to which looking behavior was biased towards the target region containing the Einstellung move. Following these analyses, we will then discuss the retrospective responses that were provided by the expert and novice chess players during the post-study interview. For all of the analyses reported below, we excluded two of the trials from the novice chess players. Specifically, we excluded one trial from Problem 2 because the chess player selected an illegal move, and we excluded another trial from Problem 3 because the chess player did not fixate on the target region.

### Analysis of Move Quality

As summarized in Appendix S1, we first examined the quality of the moves selected by the expert and novice chess players, for each of the four experimental problems (1,2,3,4). To assess move quality, we asked five expert chess players who did not participate in the study to rate each move on a scale from 1 to 10 (1 = a blunder, 10 = a very strong move). Three of these expert raters were International Masters with FIDE (World Chess Federation) ratings above 2300, and two of the raters were Grand Masters with FIDE ratings above 2500. In addition, we consulted two chess programs (Houdini 2 Pro and Deep Rybka 4). Both of these chess programs have Elo ratings of approximately 3000. Appendix S1 contains the move quality ratings (averaged across the five expert raters), the program scores (averaged across the two programs), the location of each move on the board, and the frequency with which each move was selected by the expert and novice players. Not surprisingly, as shown in Appendix S1, the experts were better able to select the optimal moves than the novices, and the experts showed superior overall performance for both of the dependent measures of move quality (i.e., expert ratings: *t*(32) = 6.04, *p*<.001; chess program scores: *t*(32) = 4.93, *p*<.001).

Most strikingly, although the experts showed superior overall performance, an equal proportion of novices and experts selected the suboptimal Einstellung move in Problem 1 (i.e., 8 out of 17 players for both groups) instead of the optimal move on the board. Thus, Problem 1 replicates prior findings that the presence of a familiar good solution can prevent chess players from choosing a better solution [7]–[8], [17], and reveals that an equal proportion of experts and novices were attracted to the Einstellung move. However, when the Einstellung move was a blunder (i.e., Problems 2, 3 and 4), all of the experts and the majority of the novices were able to avoid selecting the Einstellung move. Overall, this pattern of results supports our hypothesis that a reduction in the move quality of the familiar solution can weaken the strength of the Einstellung effect.

### Analysis of Target Region Eye Movement Measures

To further investigate the Einstellung effect, we next examined the extent to which the expert and novice players’ eye movements were directed towards the target region of the board. As a starting point for this analysis, we used the following measures (averaged across all four problems) to compare the eye movements of the expert and novices players: (1) Time to first fixation (i.e., the interval of time between the start of the trial, and the start of the first fixation on the target region); (2) Average dwell duration (a dwell is defined as one or more consecutive fixations on the target region, prior to the eyes moving to a different region of the board); (3) Total dwell time (the sum of the duration of all of the dwells on the target region); (4) Number of dwells (the total number of dwells on the target region); (5) Percentage of looking time (the proportion of time that the chess players spent looking at the target region of the board). Table 1 displays the means and standard errors of the different measures and the corresponding *t* test results.

**Table 1:** Target region eye movement measures (averaged across all four experimental problems) and corresponding t-test results, by level of expertise (expert, novice).

Measure	Expert	Novice	Difference(Novice – Expert)	Significance
Time to first fixation (ms)	407(56)	719(89)	312	*t* = 2.99, *p*<.01
Average dwell duration (ms)	2395(197)	3440(355)	1045	*t* = 2.58, *p*<.05
Total dwell time (ms)	52005(5540)	51397(6887)	−608	*t* <1
Number of dwells	27(3.5)	18(2.1)	−9	*t = *2.06, *p<*.05
Percentage of looking time	.62(.01)	.63(.02)	.01	*t* <1

Note – For the *t* tests shown above, *df* = 32.

The standard errors are shown in brackets.

As shown in Table 1, the experts displayed significantly shorter times to the first fixation on the target region, relative to the novices. This ability of the experts to rapidly fixate on the target region in the corner of the board is consistent with their previously demonstrated processing advantage for domain-related perceptual patterns in their peripheral vision ([18]–[21]; for reviews see [22]–[23]). Given that the chess players began the trial by fixating on the center of the board, it is remarkable that the chess players were able to fixate on the target region within an average of 407 ms for experts, and 719 ms for the novices. Moreover, such rapid fixations on the target region indicate that both the novice and expert players began the trial by considering the Einstellung move, which coincides with prior investigations of the Einstellung effect that showed that the familiar solution comes to mind first [7]–[8]. In addition, relative to the novices, the chess experts displayed shorter average dwell times and higher numbers of dwells in the target region. There were no significant expert/novice differences for the remaining two measures (i.e., percentage of looking time and total dwell time).

### Analysis of Looking Behaviour Over Time

Next, we examined the extent to which looking behaviour changed over time, by dividing each of the trials into four time intervals of equal length (for a similar analysis procedure, see [7], [24]). Thus, the length of these intervals varied depending on the duration on the trial, which allowed us to combine the data from trials of different durations. We then calculated the percentage of looking time and the number of dwells, for each of the time intervals (1,2,3,4), for each level of expertise (expert, novice), and for each type of Einstellung problem (i.e., suboptimal versus blunder).

#### Suboptimal move Einstellung problem

**Figure 2:**
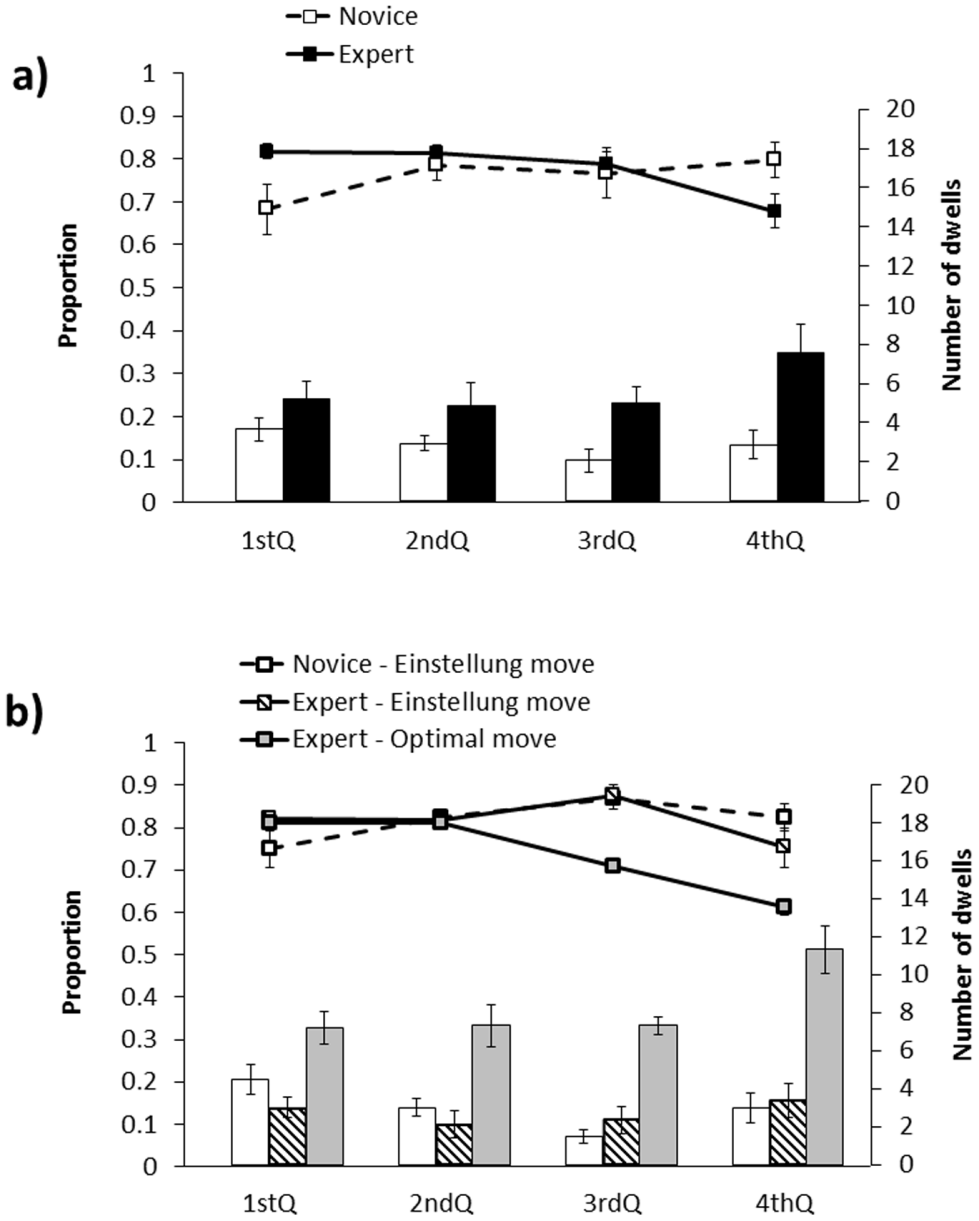
The percentage of looking time and the number of dwells in the target region in the suboptimal move Einstellung problem (i.e., Problem 1), as a function of time, for a) all expert and novice chess players, and b) the subset of expert players who selected the optimal moves on the board (i.e., Ng2 or Nc2) and the expert and novice players who selected the Einstellung move (i.e., Ba7). See text for further details.

The pattern of results for the suboptimal move problem (i.e., Problem 1) revealed expert/novice differences for both the percentage of looking time and the number of dwells measures. As shown in Figure 2 (Panel A), the percentage of looking time measure revealed that the experts spent more time in the target region than the novices during the first quarter of the trial. However, for the remaining three time intervals, the experts (but not the novices) gradually looked away from the target region. This difference in the pattern of results for experts and novices was reflected by a significant linear trend for the experts (*F*(1, 66) = 13.12, *p*<.01) but not for the novices (*F*(1, 66) = 2.25, *p = *.138), and by a significant two-way interaction between expertise and time interval (*F*(3, 96) = 3.95, *p*<.05). In addition, as shown in Figure 2 (Panel A), the number of dwells in the target region increased over time for expert players (but not the novices), as reflected by a significant interaction between expertise and time interval, (*F*(3, 96) = 4.88, *p*<.01).

However, the global expert/novice differences shown in Figure 2 (Panel A) are somewhat misleading given that there were two distinct groups of experts (i.e., the experts who selected the optimal move, and the experts who selected the Einstellung move). To test our hunch that the experts/novice differences were largely driven by the experts who chose the optimal solution, we conducted a more fine-grained analysis that contrasted the 9 experts who selected the optimal moves on the board (i.e., Ng2 or Nc2), with the 8 expert and 8 novice players who selected the Einstellung move (i.e., Ba7). As shown in Figure 2 (Panel B), this analysis replicated [7]’s findings for the percentage of looking time measure, by revealing that the chess players who chose the Einstellung move continued to fixate on this solution throughout the trial. Interestingly, the expert and novice players who chose the Einstellung move were equally unable to disengage from the target region, as indicated by a lack of linear trends for both the experts (*F <1*) and the novices (*F*(1, 30) = 1.76, *p = *.194), and by the lack of an interaction between expertise and time interval (*F*(3, 42) = 1.08, *p = *.369). In addition, there were no differences in the pattern of results for the number of dwells when we contrasted the experts and novices who selected the Einstellung move, as shown by a lack of an interaction between expertise and time interval (*F*(3, 42) = 2.34, *p* = .087).

In marked contrast, the group of experts that selected the optimal move was better able to disengage their attention from the target region, as shown by a significant linear trend (*F*(1, 34) = 43.45, *p*<.001), as well as by significant two-way interactions between move choice (optimal vs. Einstellung) and time interval when we contrasted the optimal-move experts with the Einstellung-move experts (*F*(3, 45) = 3.65, *p*<.05) and with the novices (*F*(3, 45) = 9.21, *p*<.001). Moreover, the experts who selected the optimal move had a higher number of dwells than the Einstellung-move experts (*F*(1, 15) = 12.34, *p*<.01) and novices (*F*(1, 15) = 13.46, *p*<.01), and this difference increased over time as shown by significant two-way interactions between move choice and time interval when we contrasted the optimal-move experts with the Einstellung-move experts (*F*(3, 45) = 4.19, *p*<.05) and with the novices (*F*(3, 45) = 6.60, *p*<.01). Overall, this pattern of results confirms that the expert/novice differences in Figure 2 (Panel A) were driven by the subset of experts who selected the optimal move, since the experts and novices who selected the Einstellung move did not differ from one another on either the percentage of looking time measure or the number of dwells measure.

#### Blunder move Einstellung problems

**Figure 3:**
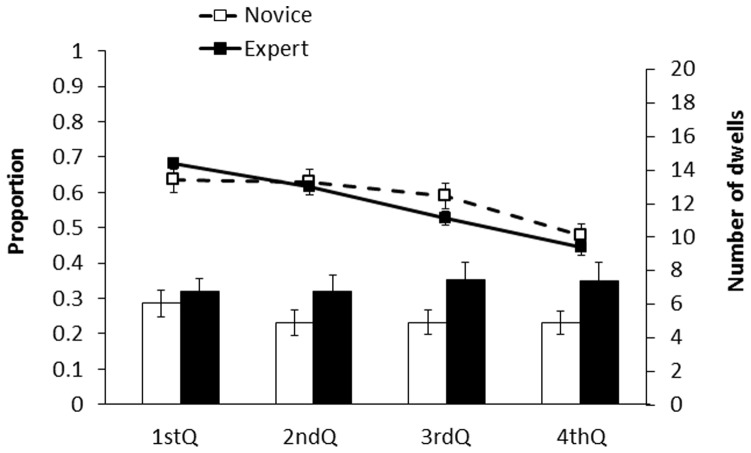
The percentage of looking time and the number of dwells in the target region in the blunder move Einstellung problems (i.e., Problems 2, 3, and 4), as a function of time, for all expert and novice chess players. See text for further details.

As can be seen from Figure 3, the pattern of results for the blunder move problems (i.e., Problems 2, 3 and 4) revealed that both the experts and novices were able to gradually disengage their attention from the target region containing the Einstellung move. Consequently, unlike in the suboptimal move problem (i.e., Problem 1), the percentage of looking time measure produced a significant linear trend for both the experts (*F*(1, 66) = 79.88, *p*<.001) and novices (*F*(1, 66) = 10.84, *p*<.01), and there were no interactions between level of expertise and time interval (*F*(3, 96) = 1.80, *p = *.152). Thus, relative to the suboptimal move problem, both the experts and novices were better able to resist the Einstellung effect for the blunder move problems, as shown by their greater ability to disengage their attention from the target region (see Figure 3) and the fact that all of the experts and the majority of the novices avoided choosing the Einstellung move (see Appendix S1). Overall, this pattern of results supports our hypothesis that the Einstellung effect would be weakened when the Einstellung move was a blunder (i.e., Problems 2, 3, and 4) rather than an advantageous but suboptimal move (i.e., Problem 1).

Finally, similar to the suboptimal move problem, the number of dwells in the target region increased over time for the experts but not for the novices, as shown by a significant interaction between level of expertise and time interval (*F*(3, 96) = 4.03, *p*<.05). Thus, as shown in Table 1 and Figures 2 and 3, the number of dwells measure revealed a consistent qualitative difference in the pattern of looking behaviour for the experts versus novices, such that the experts displayed shorter and more frequent dwells on the target region as the trial progressed.

### Retrospective Responses

To further explore the chess player’s problem-solving strategies, we also examined the expert and novice players’ retrospective responses for both types of Einstellung problem (i.e., suboptimal versus blunder). For both types of problems, the majority of the chess players stated that they considered the Einstellung move first, which coincides with our findings of rapid times to the first fixation on the target region (see Table 1). However, consistent with the pattern of results for the accuracy and eye tracking measures, both the expert and novice chess players had more difficulty ruling out the Einstellung solution when it was a suboptimal move rather than a blunder move. In fact, out of the eight expert and eight novice players who selected the suboptimal Einstellung move (i.e., “Ba7”), two of the experts and four of the novices did not rule out checkmate (Sample expert comment: “I was actually wrong to think that Ba7 leads to checkmate in this position”; Sample novice comment: “By moving the bishop in, it is a checkmate”). The remaining players thought that the suboptimal Einstellung move would improve white’s position (Sample expert comment: “No forced checkmate that I can see…After Ba7 Na7 Qa7 Kc8 Qc5 white looks to have improved its position [by] giving the queen more mobility ….”; Sample novice comment: “I chose to move bishop to a7 because….white can continue attacking with black having less defenders”), although several of the experts were unsure if it was the best move (e.g., “I’m unclear as to if Ba7 is best but it looks promising”). In contrast, the nine experts who chose the optimal move stated that they ruled out checkmate in the target region, and then considered the optimal move outside of the target region (e.g., “I didn’t see the mate on a7, so I looked at the other side of the board…”). Some of these experts considered the long-term consequences of the optimal move for the pieces within the target region (e.g., “The only piece missing in action was N on e3, so I wanted to bring it in by Nc2-b4 then possibly Na6”), which might account for why the experts who chose the optimal move showed an increase in the number of dwells in the target region, relative to the experts and novices who selected the Einstellung move. Finally, unlike the suboptimal move Einstellung problem, the retrospective responses for the blunder move Einstellung problems revealed that all of the experts and the majority of the novice players ruled out the Einstellung moves as a viable option (Sample expert comment: “Though it looks like white has an attack, Black is defending it well”; Sample novice comment: “I was not able to find a good move in the dotted region of the board”).

## Discussion

The present findings revealed new insights concerning the processes underlying the Einstellung (mental set) effect, in which a familiar solution blocks the discovery of a better solution [1]. Most importantly, the subset of expert and novice chess players who chose the familiar but suboptimal Einstellung move continued to look at this move throughout the trial – even though there was an optimal move located in a different region of the board – whereas the experts who discovered the optimal move were able to gradually disengage their attention from the Einstellung move. This pattern of results replicates [7], using a choose-a-move task that employed a single problem to elicit both the optimal and suboptimal move choices, rather than requiring two different versions of the problem as in [7]. Thus, our findings provide convergent evidence for [7]’s conclusion that the Einstellung effect operates by biasing the problem-solvers’ attention towards problem features that are associated with the familiar solution, thereby preventing the discovery of new solutions. In the present study, this bias in attention towards the familiar solution was evident for both the experts and the novices who chose the Einstellung move, which underscores prior findings that the Einstellung effect is pervasive across a wide range of levels of expertise [8].

Extending [7]–[8], we also uncovered a key boundary condition of the Einstellung effect, by showing that the magnitude of the Einstellung effect was severely reduced when we introduced a new type of Einstellung move that was a clear blunder rather than an advantageous (but suboptimal) move. Specifically, unlike the suboptimal Einstellung move in Problem 1, all of the experts and the majority of the novices were able to avoid choosing the blunder moves in Problems 2, 3, and 4, and both the expert and novice chess players were able to gradually disengage their attention from the target region containing the blunder move. These findings shed light on the boundary conditions of the Einstellung effect, by revealing that the outcome of the Einstellung move (suboptimal versus blunder) plays a critical role.

One possible explanation for why the blunder moves reduced the Einstellung effect is that the blunder moves provided feedback that the familiar solution was not viable. This type of feedback may have improved performance on the blunder move Einstellung problems by providing the chess players with increased motivation to search for a new solution. In contrast, such feedback was not available for the suboptimal move Einstellung problem, because the suboptimal move was advantageous for white. Moreover, similar to the suboptimal move, the longer checkmate solution in [7] might have given chess players the impression that the problem was already solved, which could have reduced their motivation to find a new solution. Thus, the Einstellung effect may be especially pernicious when problem-solvers are not given feedback that they are using a suboptimal strategy (for a related discussion, see [13], [25]).

In addition, another implication of the present findings is that the percentage of looking time measure employed by [7] is not always sufficient, and should be supplemented with additional measures, such as the number of dwells measure. This is because the percentage of looking time measure alone cannot reveal whether target region fixations were due to an inability to rule out the Einstellung move, or due to long-term strategizing concerning how the optimal move would impact the pieces within the target region. To the extent that the chess players were returning to the target region to strategize about the impact of the optimal move, then the percentage of looking time measure could be over-estimating the chess players’ inability to disengage from the Einstellung move. In the present study, the number of dwells measure seemed to provide a good index that this type of optimal move strategizing was occurring, because the experts who discovered the optimal move displayed shorter and more frequent dwells in the target region, relative to the experts and novices who remained fixated on the suboptimal Einstellung move. Moreover, for the blunder Einstellung problems, the experts showed shorter and more frequent dwells than the novices, even though the blunder move problems did not reveal any expertise differences for the percentage of looking time measure. This pattern of results underscores the importance of supplementing the percentage of looking time measure with additional measures, to provide a more complete understanding of why chess players are fixating on a particular region of the board.

Finally, future work could investigate the extent to which the mechanisms underlying the Einstellung effect in chess are related to other thinking errors beyond the chess domain. More specifically, as discussed by [6]–[8], the chess players’ bias in attention towards the familiar checkmate solution might reflect a more general cognitive tendency to selectively focus attention on information that is associated with an already activated knowledge schema. To give an example, this mechanism could be contributing to the satisfaction of search (SOS) effect that has been studied extensively in the domain of medical expertise [26]–[29]. The SOS effect refers to the finding that the discovery of one abnormality can prevent expert radiologists from discovering additional abnormalities. Although the mechanisms underlying SOS are controversial, one possibility is that the discovery of an obvious abnormality could subsequently bias attention towards visual features that are related to this type of abnormality, rather than towards features that are associated with more subtle abnormalities [30]. Moreover, beyond the domain of visual expertise, this bias in attention towards already activated knowledge schemas could be contributing to the tendency of political experts and scientists to ignore evidence that does not fit with their existing theories [31]–[32], as well as memory findings that it is difficult to recall details that do not fit with already-activated knowledge schemas (i.e., the part-set cuing phenomena: [33]–[34]). Future work could continue to explore the extent to which thinking errors in different domains and tasks are potentially driven by common mechanisms.

## Supporting Information

Appendix S1 **For each of the four experimental problems (1,2,3,4), Appendix S1 contains the move quality ratings (averaged across the five expert raters), the program scores (averaged across the two programs), the location of each move on the board (1 = inside the target region, 0 = outside the target region), and the frequency with which each move was selected by the expert and novice players.** See text for further details.
(DOCX)
