# Supplementary material for: The Mechanisms and Boundary Conditions of the Einstellung Effect in Chess: Evidence from Eye Movements
Source: PLoS One. 2013 Oct 4;8(10):e75796. doi: 10.1371/journal.pone.0075796 (PMC3790829; doi:10.1371/journal.pone.0075796)
Supplement: Appendix S1 — For each of the four experimental problems (1,2,3,4), Appendix S1 contains the move quality ratings (averaged across the five expert raters), the program scores (averaged across the two programs), the location of each move on the board (1 = inside the target region, 0 = outside the target region), and the frequency with which each move was selected by the expert and novice players. See text for further details. (DOCX) [file pone.0075796.s001.docx]

Appendix S1

*Move location, move quality rating, chess program score, and move selection frequency by experts and novices, for each problem and each move. See text for further details.*

|  | Location of move  (inside/outside target region) | | Move Quality | | Move selection frequency (number of players) | |
| --- | --- | --- | --- | --- | --- | --- |
| Move | Initial  square | Destination square | Rating by human experts | Program score | Expert | Novice |
|  |  |  |  |  |  |  |
| Problem 1 |  |  |  |  |  |  |
| Ng2  Nc2  Ba7*  Na7  Nc7  Bd6  Nc4  Bb6 | 0  0  1  1  1  1  0  1 | 0  0  1  1  1  1  0  1 | 9  6.8  5  4.4  1.2  1  1  1 | 1.76  0.585  1.03  0.435  -0.62  -2.77  -3.63  -4.025 | 3  6  8  0  0  0  0  0 | 0  1  8  3  2  1  1  1 |
| Avg. Move Quality: rating (Expert: 6.34, Novice: 3.85, *t*(32) = 4.21, *p* <.001),  program score (Expert: 1.00, Novice: -.09, *t*(32) = 2.55, *p* <.05) | | | | | | |
| Problem 2 |  |  |  |  |  |  |
| Na3  Rf3  Ne1  Rf4  Nd5  Kg1  Ng6  f7  Qg7* | 0  0  0  0  1  0  1  1  1 | 0  0  0  0  0  0  1  1  1 | 9.2  7.4  6.8  6.6  4.8  4.6  2  1.4  1 | 5.45  2.31  2.055  1.43  0.17  2.135  -1.25  -3.215  -10.67 | 3  7  5  2  0  0  0  0  0 | 3  0  0  0  4  2  1  3  3 |
| Avg. Move Quality: rating (Expert: 7.45, Novice: 4.08, *t*(31) = 4.43, *p* <.001),  program score (Expert: 2.69, Novice: -1.35, *t*(31) = 2.97, *p* <.01) | | | | | | |
| Problem 3 |  |  |  |  |  |  |
| Rg5  b3  Qb4  Rc1  c4  Rh1  Nd6  Na7  Na8  Qa8*  Qa7* | 0  0  1  0  0  0  1  1  1  1  1 | 0  0  0  0  0  0  0  1  1  1  1 | 8.2  7  5.8  5.4  5.2  4.8  3.2  1.6  1.4  1  1 | 2.585  0  0  0.295  0.79  0  0.045  -2.875  -1.94  -12.62  -12.985 | 11  1  0  0  3  0  2  0  0  0  0 | 2  2  1  2  0  1  2  1  3  0  2 |
| Avg. Move Quality: rating (Expert: 7.01, Novice: 4.13, *t*(31) = 3.64, *p* <.01),  program score (Expert: 1.82, Novice: -1.80, *t*(31) = 3.15, *p* <.01) | | | | | | |
|  | | | | | | |
| Problem 4 |  |  |  |  |  |  |
| Rb3  Rd1  a3  Ne7  Ng3  Ng8  Rf7  Nf7*  Rg8* | 0  0  0  1  1  1  1  1  1 | 0  0  0  0  0  1  1  1  1 | 9.2  8.2  5.8  4  2.6  1.2  1  1  1 | 1.18  0.23  0.445  -0.42  -1.18  -5.195  -5.78  -3.18  -7.56 | 11  3  0  1  1  1  0  0  0 | 9  2  2  2  1  0  1  0  0 |
| Avg. Move Quality: rating (Expert: 7.86, Novice: 7.20, *t* < 1),  program score (Expert: .40, Novice: .25, *t* < 1) | | | | | | |
|  | | | | | | |
|  |  |  |  |  |  |  |

Note – The moves shown above are denoted as follows: *move associated with Einstellung solution.
